# MRI of Acute Low Back Pain: About an Uncommon Pitfall

**DOI:** 10.7759/cureus.22905

**Published:** 2022-03-06

**Authors:** Meriem Ben Ghanem, Niloufar Sadeghi, Stéphanie Elens

**Affiliations:** 1 Radiology, Erasmus Hospital, Brussels, BEL; 2 Radiodiagnosis, Erasmus Hospital, Brussels, BEL

**Keywords:** excluded disc fragment, inflammatory disc herniation, lumbago, epidural mass, posteriorly migrated disc extrusion

## Abstract

Posteriorly migrated disc extrusion may mimic tumoral masses on MRI with contrast; still, this diagnosis must be evoked in patients presenting acute low back pain with a posterior epidural mass.

We describe a case of epidural posterior migration of an inflammatory lumbar disc herniation in a young patient with acute lumbosciatica. MRI showed an intracanalar mass with intense global enhancement, which is an uncommon feature of this rare condition.

## Introduction

Migration of an excluded or non-excluded disc herniation usually occurs into the anterior epidural space. Posterior migration of excluded disc fragments is an extremely rare entity and is a radiological challenging diagnosis. The differential diagnosis includes neoplastic masses that may lead to unnecessary surgical excision in case of misdiagnosis [1-4].

We report a case of dorsally sequestrated lumbar disc herniation that was initially diagnosed as a meningioma on MRI. We then analyze the clinical and imaging features in light of literature data.

## Case presentation

**Figure 1:**
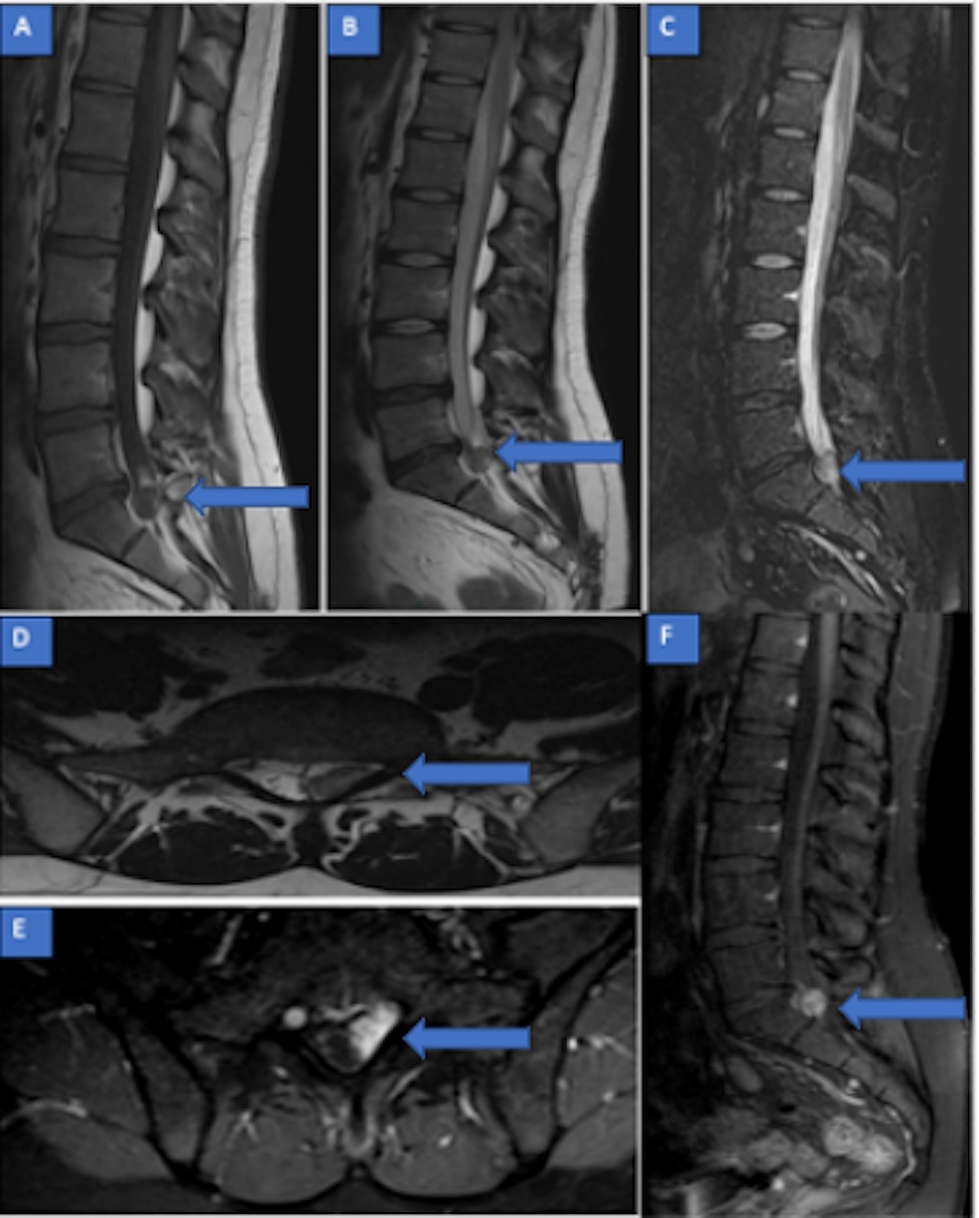
Lumbar spine MRI (November 2019) (A) Sagittal T1, (B) T2, (C) T2 STIR, (D): axial T2: intracanalar expansive mass of 14 mm, located in the left posterolateral epidural space at the S1 level, hypointense on T1-WI, heterogeneous on T2-WI and hyperintense on T2 STIR-WI. (E, F) Axial and sagittal T1 with gadolinium: homogeneous and intense enhancement of the whole lesion. MRI - Magnetic Resonance Imaging

A 28-year-old man was admitted to the emergency department with chronic low back pain. He reported acute sciatica irradiating to the left lower limb for three days, exacerbated by movement, not resolving with usual analgesic treatments. He had no sensory-motor deficits and the physical examination was normal except for a left Lasègue sign. An MRI was prescribed followed by an appointment for a neurosurgical consultation within two weeks. The MRI revealed a minor discopathy and an intracanalar expansive mass, measuring 14 mm of major axis, located in the left posterolateral epidural space. It was displacing the dural sac and the left S1- S2 roots to the right. Because of this contact with the left lamina of S1 and the intense enhancement after contrast injection, the diagnosis made was meningioma (Figures 1A-1F). 

**Figure 2:**
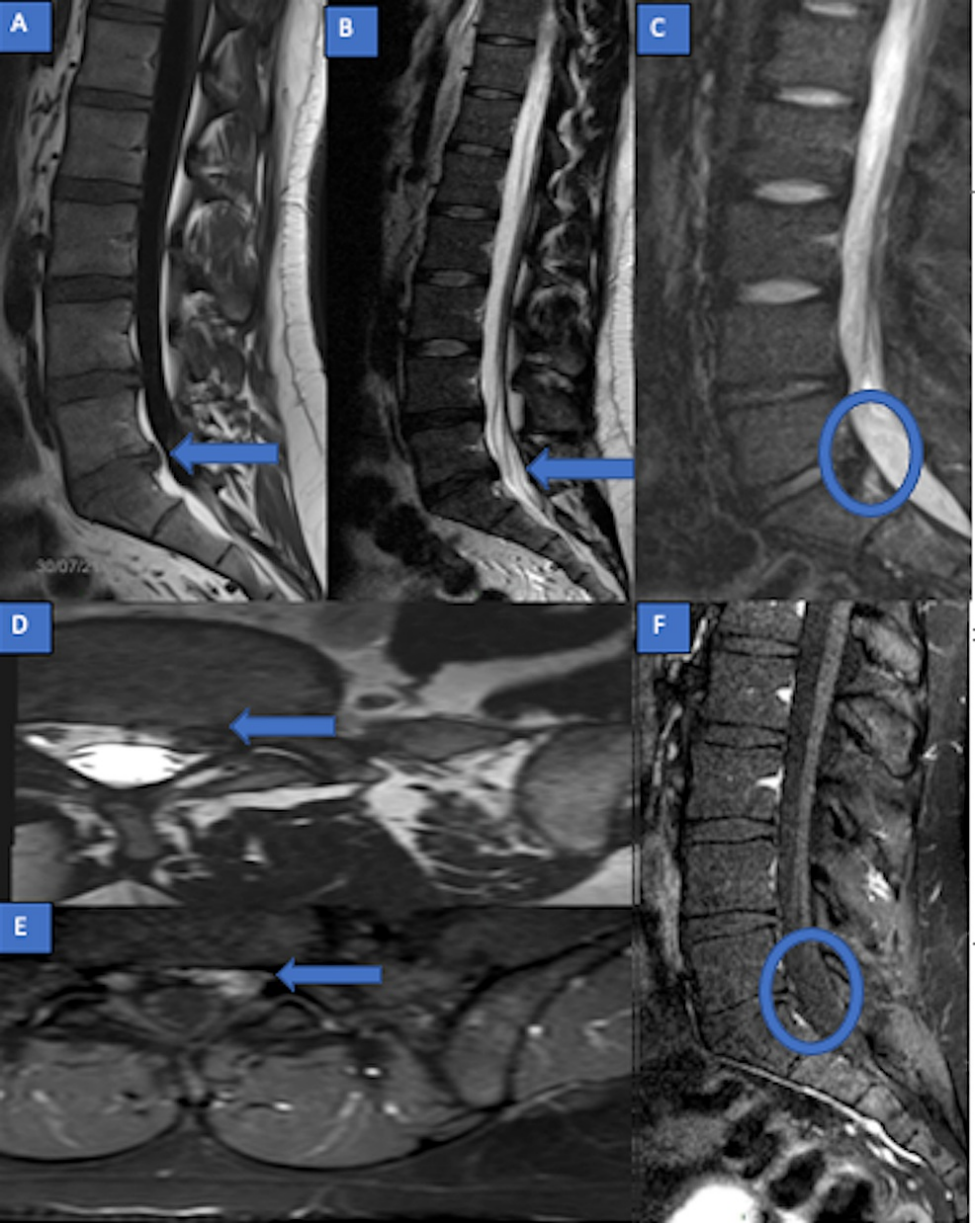
Lumbar spine MRI (February 2021) (A) Sagittal T1, (B) T2, (C) T2 STIR; (D) axial T2; (E, F): axial and sagittal T1 post-contrast: posterior descending discal protrusion, in conflict with the left S1 root. There is no intracanalar mass. MRI - Magnetic Resonance Imaging

The patient lost to follow-up and no additional support was carried out until he was referred by his doctor for a recurrence of low back pain after a year. The control MRI no longer shows the previously described intracanal mass with intense enhancement in L5-S1 (Figures 2A-2F). We deduce that the disappeared mass was actually an inflammatory discal extrusion that has resolved. We also note a persistent discopathy on L4-L5 and L5-S1. 

## Discussion

Anatomically, migration of an excluded disc fragment usually occurs in the anterior epidural space and is sub-ligamentary. The posterior longitudinal ligament includes a medial septum which subdivides the anterior epidural space into a right and left space, thus providing an anatomical barrier to right-left disc migration. In addition, the lateral membranes that close this anterior epidural space and the spinal nerves represent two further barriers to anteroposterior migration [1,2]. Therefore, excluded disc herniation with posterior migration is an uncommon event with a frequency of less than 1% [3]. The most frequent locations are L3-L4 and L4-L5 [1]. Classically reported clinical presentations are radiculalgia and cauda equina syndrome [1,3]. The differential diagnosis includes tumors, mainly schwannoma and meningioma, synovial cyst, epidural abscess, and hematoma [1-5]. MRI is the recommended examination before a potential decompression surgery with laminectomy and discectomy [1,3]. On MR scans, the sequestrated disc usually shows a low signal intensity on T1-WI and homogeneous or heterogeneous hyper-intensity on T2-WI. It may also be hypo-intense due to disc degeneration and water loss on T2-WI. After injection of gadolinium, the central part of the disc fragment does not take the contrast while the periphery is enhanced. This peripheral enhancement reflects the inflammatory response and is a key element in the positive diagnosis of herniation as opposed to tumors, where contrast enhancement is generally uniform [1-4]. However, global enhancement of the fragment, although infrequent, has been reported in 14% of cases. In the literature, imaging did not suggest a posteriorly migrated herniated disc in 68% of cases and the diagnosis could only be made pre-operatively [1].

## Conclusions

Excluded disc fragment might be considered in cases with a lumbar posterior epidural mass in a patient presenting with acute lumbago. MRI with gadolinium is the best diagnostic tool, with a classically peripheral enhancement of the disc fragment. However, in the case of severe inflammatory disc herniation, a complete enhancement of the disc fragment would constitute a pitfall and this diagnosis should not be excluded.
